# Loss of Interneuron-Derived Collagen XIX Leads to a Reduction in Perineuronal Nets in the Mammalian Telencephalon

**DOI:** 10.1177/1759091416689020

**Published:** 2017-01-01

**Authors:** Jianmin Su, James Cole, Michael A. Fox

**Affiliations:** 1 Virginia Tech Carilion Research Institute, Roanoke, VA, USA; 2 Department of Biological Sciences, Virginia Tech, Blacksburg, VA, USA; 3 Department of Pediatrics, Virginia Tech Carilion School of Medicine, Roanoke, VA, USA

**Keywords:** aggrecan, cerebral cortex, collagen, extracellular matrix, hippocampus, perineuronal net

## Abstract

Perineuronal nets (PNNs) are lattice-like supramolecular assemblies of extracellular glycoproteins that surround subsets of neuronal cell bodies in the mammalian telencephalon. PNNs emerge at the end of the critical period of brain development, limit neuronal plasticity in the adult brain, and are lost in a variety of complex brain disorders diseases, including schizophrenia. The link between PNNs and schizophrenia led us to question whether neuronally expressed extracellular matrix (ECM) molecules associated with schizophrenia contribute to the assembly of these specialized supramolecular ECM assemblies. We focused on collagen XIX—a minor, nonfibrillar collagen expressed by subsets of telencephalic interneurons. Genetic alterations in the region encoding collagen XIX have been associated with familial schizophrenia, and loss of this collagen in mice results in altered inhibitory synapses, seizures, and the acquisition of schizophrenia-related behaviors. Here, we demonstrate that loss of collagen XIX also results in a reduction of telencephalic PNNs. Loss of PNNs was accompanied with reduced levels of aggrecan (Acan), a major component of PNNs. Despite reduced levels of PNN constituents in collagen XIX-deficient mice (*col19a1^−^^/^^−^)*, we failed to detect reduced expression of genes encoding these ECM molecules. Instead, we discovered a widespread upregulation of extracellular proteases capable of cleaving Acan and other PNN constituents in *col19a1^−^^/^^−^* brains. Taken together, these results suggest a mechanism by which the loss of collagen XIX speeds PNN degradation and they identify a novel mechanism by which the loss of collagen XIX may contribute to complex brain disorders.

## Introduction

Extracellular matrix (ECM) is a noncellular component of all tissues, composed of a dense mixture of proteins and polysaccharides. Outside of the nervous system, major constituents of ECM include laminins, fibrillar and nonfribrillar (i.e., unconventional) collagens, fibronectins, nidogens, tenascins, and proteoglycans (i.e., proteins with one or more covalently linked glycosaminoglycan side chains) (LeBleu et al., 2007; Frantz et al., 2010). Interactions between these ECM constituents lead to their assembly into supramolecular structures, which include basal laminas, basement membranes, filaments, and fibrils. These supramolecular ECM structures provide structural support for adjacent cells, act as reservoirs for growth factors, morphogens, and extracellular proteases, and impact nearly all biological functions outside of the brain.

Despite our vast knowledge of ECM assemblies in most tissues, our understanding of their role in brain development and function remains limited. This is surprising given that a significant portion of brain parenchyma comprises extracellular space. In the juvenile brain, when neural circuits are still being assembled, extracellular space accounts for ∼40% of brain parenchyma (Nicholson and Sykova, 1998). Although there is a dramatic reduction in the volume of extracellular space as the mammalian brain matures, a significant fraction of adult brain parenchyma remains occupied by extracellular space (∼20%; Nicholson and Sykova, 1998; Sykova and Nicholson, 2008; Korogod et al., 2015; Pallotto et al., 2015). Despite this, there has been little attention paid to the presence and roles of supramolecular ECM assemblies in the mammalian brain. This likely stems from a general lack of fibrillar assemblies of ECM constituents in the healthy brain and a limited presence of basal laminas or basement membranes. Basal lamina-like structures in the brain are limited to vascular structures and to “fractones,” specialized ECM assemblies associated with the subventricular zone (Mercier et al., 2002; Mercier, 2016). However, it is becoming increasing clear that many ECM constituents are in fact generated by neurons and glia in the developing, adult, and injured brain and these constituents play essential roles in neuronal migration, neural circuit formation, and synaptic plasticity (Dityatev et al., 2010; Dityatev and Rusakov, 2011; Frischknecht and Gundelfinger, 2012; Risher and Eroglu, 2012; Heikkinen et al., 2014; Smith et al., 2015). But, at present, we lack a clear understanding of how these factors are assembled in the extracellular space surrounding neurons and glia in the brain. The one exception to this is the perineuronal net (PNN)—a specialized, lattice-like supramolecular assembly of ECM constituents that surrounds the cell bodies and proximal neurites of select classes of interneurons (Celio and Blumcke, 1994; Celio et al., 1998; Yamaguchi, 2000). PNNs emerge late in postnatal brain development, coinciding with closing of the critical period of brain development. For this reason it has been hypothesized that these specialized matrices limit the neuroplastic potential of adult brains (Rhodes and Fawcett, 2004; Faissner et al., 2010; Wang and Fawcett, 2012). PNNs have garnered attention not only for their potential role in developmental plasticity but also because they appear disrupted in a number of psychiatric and complex brain disorders.

Interestingly, the molecular composition of PNNs differs from supramolecular assemblies of ECM constituents outside the brain. PNNs are composed primarily of lecticans (a family of chondroitin sulfate proteoglycans [CSPGs]), tenascins, hyaluranonin and proteoglycan binding link proteins (HAPLNs), and hyaluran (Zimmermann and Dours-Zimmermann, 2008; Frischknecht and Seidenbecher, 2012; Morawski et al., 2012). More traditional ECM constituents, such as laminins, nidogens, fibronectin, and fibrillar and nonfibrillar collagens, are either absent from PNNs or their roles remain unclear. However, we have recently become interested in the link between collagen XIX and PNNs. Collagen XIX is a nonfibrillar, unconventional collagen generated by telencephalic interneurons in the postnatal rodent brain (Sumiyoshi et al., 1997; Su et al., 2010; Su et al., 2016). Collagen XIX-expressing neurons are present in regions densely packed with PNNs. And, just as PNNs have been linked to schizophrenia and complex brain disorders (Berretta, 2012; Berretta et al., 2015; Bitanihirwe et al., 2016), genetic alterations in the region encoding collagen XIX have been associated with schizophrenia (Liao et al., 2012). Furthermore, targeted deletion of collagen XIX in mice leads to the acquisition of schizophrenia-related behaviors and seizures (Su et al., 2016). Based upon these similarities, we tested whether collagen XIX contributes to the assembly or maintenance of PNNs by assessing PNN morphology and distribution in targeted mouse mutants lacking this collagen (*col19a1^−^^/^^−^*). Our data revealed a significant reduction in the number of PNNs in regions of mouse brain that normally generate collagen XIX. Loss of PNNs was accompanied by a widespread reduction in protein levels of lecticans in these regions of *col19a1^−^^/^^−^* mutant brains. However, we failed to detect reduced levels of lectican or PNN constituent mRNA in mutants, suggesting that the reduced number of PNNs in collagen XIX-deficient brains did not result from diminished lectican transcription. Finally, we tested whether the loss of this unconventional neuronally expressed collagen altered the expression of extracellular proteases that degrade lecticans and PNNs. To our surprise, we discovered a widespread upregulation in the transcription of matrix metalloproteinases (MMPs) and a distintegrin and metalloproteinase with thrombospondin motif proteases (ADAMTSs) in these regions of *col19a1^−^^/^^−^* mutant brains.

## Materials and Methods

### Animals

CD1 and C57BL/6 mice were obtained from Charles River Laboratories (Wilmington, MA, USA). The generation of collagen XIX null mice (*col19a1^−^^/^^−^*; previously referred to as N19) was previously described (Sumiyoshi et al., 2004). Col19a1*^−^^/^^−^* mice were backcrossed for more than 10 generations on C57BL/6 mice. *Parv-cre* and *thy1-stop-yfp* (line 15) mice were obtained from Jackson Laboratories (stock numbers 008069 and 005630, respectively). Genomic DNA was isolated from tail using the HotSHOT method (Truett et al., 2000), and genotyping was performed with the following primers: lacZ 5′-TTC ACT GGC CGT CGT TTT ACA ACGTCG TGA-3′ and 5′-ATG TGA GCG AGT AAC AAC CCG TCG GAT TCT-3′; col19a1 (exon4) 5′-CTTCGC AAA ACG CAT GCC TCA GA-3′ and 5′-TTG TTC GTT TGT TTG TTT TTA ATC AAT CAA-3′; yfp 5′-AAG TTC ATC TGC ACC ACC G-3′ and 5′-TCC TTG AAG AAG ATG GTG CG; cre 5′-TGC ATG ATC TCC GGT ATT GA-3′ and 5’-CGT ACT GAC GGT GGG AGA AT-3′. The following cycling conditions were used on an Eppendorf Mastercycler EP: 95℃ for 5 min, followed by 35 cycles of amplification (95℃ for 30 s, 52℃ for 30 s, and 72℃ for 45 s), and 10 min at 72℃. All analyses conformed to National Institutes of Health (NIH) guidelines and protocols, approved by the Virginia Polytechnic Institute and State University Institutional Animal Care and Use Committees.

### Reagents and Antibodies

The following reagents and antibodies were purchased: Biotinylated Wisteria Floribunda Lectin (diluted 1:1000 for IHC; Vector Laboratories, Burlingame, CA, USA), mouse anti-CSPG protein core epitope (Cat315, aggrecan [Acan]; Matthews et al., 2002; Brooks et al., 2013) (diluted 1:5000 for IHC; 1:10000 for WB; Millipore, Billerica, MA), mouse anti-actin (diluted 1:10000 for WB; Millipore, Billerica, MA), rabbit anti-GFP (diluted 1:500; Life Technologies, Carlsbad, CA), rabbit anti-ADAMTS4 (diluted 1:1000 for WB; Abcam, Cambridge, MA), mouse anti-NeuN (diluted 1:100 for IHC; Millipore, Billerica, MA), and Alexa Fluor-488 Streptavidin conjugate (diluted 1:1000 for IHC; Life Technologies, Carlsbad, CA). All peroxidase-conjugated anti-mouse, -rabbit, or -sheep antibodies were from Jackson ImmunoResearch Inc. (diluted 1: 5000 for WB), and all fluorescent secondary antibodies for IHC were from Life Technologies (diluted 1:1000). All other reagents are from Fisher unless otherwise noted.

### Immunohistochemistry

Fluorescent immunohistochemistry (IHC) was performed on 16 µm cryosectioned paraformaldehyde (PFA)-fixed brain tissue as described previously (Fox et al., 2007; Su et al., 2012). Briefly, tissue slides were allowed to air dry for 15 min before being incubated with blocking buffer (2.5% normal goat serum, 2.5% bovine serum albumin, and 0.1% Triton X-100 in PBS) for 30 min. Primary antibodies were diluted in blocking buffer and incubated on tissue sections for overnight at 4℃. On the following day, tissue slides were washed in PBS and secondary antibodies diluted 1:1000 in blocking buffer were applied to slides for 1 hr at room temperature. After thoroughly washing in PBS, tissue slides were coverslipped with VectaShield (Vector Laboratories, Burlingame, CA, USA). Images were acquired on a Zeiss Examiner Z1 LSM 710 confocal microscope or a Zeiss LSM 700 confocal microscope (Oberkochen, Germany). When comparing between genotypes, images were acquired with identical parameters and analyzed blind. A minimum of three animals (per genotype and per age) were compared in all IHC experiments. IHC images were analyzed and quantified as previously described (Su et al., 2010; Levy et al., 2015; Su et al., 2016). For PNN counts in hippocampus, all images were obtained with identical parameters, and to ensure similar regions were analyzed, we only analyzed coronal sections of hippocampus that also contained central regions of visual thalamus—which includes both dorsal and ventral lateral geniculate nuclei (dLGN and vLGN, respectively). For quantification purposes, all WFA and Acan-labeled PNNs in the entire hippocampus of these sections were counted manually and at least four sections were analyzed per animal (from both hemispheres; *n* = 4 animals per genotype). Limiting analysis to identical regions of hippocampus (and identically sized hippocampi) reduced potential errors from counting different sized regions of hippocampi. Likewise, all images and analysis of PNN distribution in cerebral cortex were obtained from region- and size-matched sagittal sections of wild-type (WT) and littermate mutant mice. PNNs is all layers of motor cortex (mCtx), prefrontal cortex (pfCtx), and primary visual cortex (vCTX) were counted in sagittal sections from mutants and controls (*n* = 4 animals per genotype). Cortical regions in mutant and control sections were identified blind to the genotype using the Allen Brain Atlas references atlas (http://mouse.brain-map.org/static/atlas). Quantified data were analyzed with a Student’s *t*-test to determine statistical significance.

### Western Blot

Mouse brains were perfused with PBS, tissue was dissected in ice-cold PBS, and tissue was lysed in modified loading buffer containing 50 mmol/L Tris-HCl, pH 6.8, 2% sodium dodecyl sulfate (SDS), 10% glycerol, and protease inhibitors (1 mmol/L PMSF). Samples were homogenized, boiled for 10 min, centrifuged at 12,000 g for 10 min, and insoluble material was removed. Equal amounts of protein were loaded and separated by SDS-PAGE and transferred to PVDF membranes (Bio-Rad). SDS-PAGE was performed with 7% polyacrylamide gels (7% polyacrylamide [Fisher], 0.1% SDS [Fisher], 0.05% ammonium persulfate [APS; Fisher], 0.001% N,N,N′,N′-tetramethylethylene-diamine [TEMED; Bio-Rad] in 375 mM Tris-HCl pH 8.8) prepared with 4% stacking gels (4% polyacrylamide, 0.1% SDS, 0.05% APS, 0.001% TEMED in 125 mM Tris-HCl pH 6.8). After blocking in 5% nonfat milk in PBS (containing 0.05% Tween20), PVDF membranes (Bio-Rad, Hercules, CA) were incubated with appropriate primary antibodies, followed by HRP-conjugated secondary antibodies. Immunoblotted proteins were detected with the enhanced chemiluminescent detection system (ECL Plus, Amersham Pharmacia Biotech, Piscataway, NJ) and a Gel-Doc imaging system (Bio-Rad). Densitometry of images was performed in ImageJ, and all quantified data were normalized to actin levels.

### Quantitative PCR

RNA was isolated using the BioRad Total RNA Extraction from Fibrous and Fatty Tissue kit (BioRad, Hercules, CA). cDNAs were generated from 500 ng of RNA with the Superscript II Reverse Transcriptase First Strand cDNA Synthesis kit (Invitrogen, La Jolla, CA). The following primers were used: *acan*-qF: 5′ CCC TCA GAG TCA CAA AGA CCA 3′, *acan*-qR: 5′ TTC GCA GGG ATA AAG GAC TG 3′; *bcan*-qF: 5′ AAC ATA GGC AGC GGA AAC C 3′,* bcan*-qR: 5′ GTG GAG TGG CTG TGG CTC 3′; *tnr*-qF: 5′ AAT TTC TTG GGT CAG GGA GG 3′, *tnr*-qR: 5′ GAA CCG ACT GTG CTA AGG CT 3′; *hapln1*-qF: 5′ ATC AGC ACC AGG AGA AGC AG 3′, *hapln1*-qR: 5′ CTC CAG CTC CCA GCT AAG TG 3′; *hapln2*-qF: 5′ CTG CTT GGC ATG ATG GTG T 3′, *hapln2*-qR: 5′ CTC GAT TTG CCT CTG AGG AG 3′; *hapln3*-qF: 5′ ACA AGG AAC AGC AGG CTC AT 3′, *hapln3*-qR: 5′ CGA AGA GCT TTC TGG GGA C 3′; *hapln4*-qF: 5′ GGG TGA TAG GGG AAG ACC AC 3′; *hapln4*-qR: 5′ GTC ACC CTT CAG GAC TAC GG 3′; *has1*-qF: 5′ AAG ATG ATC GTG AGT GCT CG 3′, *has1*-qR: 5′ GAG AGA ATC CAG GAG GAC CC 3′;* has2*-qF: 5′ ACA GAT GAG GCA GGG TCA AG 3′, *has2*-qR: 5′ TGGG GTG GAA AGA GAG AAG T 3′; *has3*-qF: 5′ AGC ACT ACC AGG GCA AAC AG 3′*; has3*-qR: 5′ CCC TTG GCG TTC AGA AGA T 3′; *mmp3*-qF: 5′ AGC CTT GGC TGA GTG GTA GA 3′, *mmp3*-qR: 5′ CGA TGA TGA ACG ATG GAC AG-3′; *mmp7*-qF: 5′ GCA TTT CCT TGA GGT TGT CC 3′, *mmp7*-qR: 5′ CAC ATC AGT GGG AAC AGG C 3′; *mmp8*-qF: 5′ AGA CCG GAA TTG ATT GCT TG 3′, *mmp8*-qR: 5′ CCC AGT ATC TGA ACA CCT GGA 3′;* mmp9*-qF: 5′ CTG TCG GCT GTG GTT CAG T-3′, *mmp9*-qR: 5′ AGA CGA CAT AGA CGG CAT CC 3′; *mmp12*-qF: 5′ TTT GGA TTA TTG GAA TGC TGC 3′,* mmp12*-qR: 5′ ATG AGG CAG AAA CGT GGA CT 3′;* mmp13*-qF: 5′ GGT CCT TGG AGT GAT CCA GA 3′*; mmp13*-qR: 5′ TGA TGA AAC CTG GAC AAG CA 3′; *adamts1*-qF: 5′ GGA CAC AAA TCG CTT CTT CC 3′, *adamts1*-qR: 5′ CAG AAC ACC CGG AAC CAG T 3′; *adamts4*-qF: 5′ GTC ATG GCT CCT GTC ATG G 3′, *adamts4*-qR: 5′ CCG GTT TGT CTA AGA GGC AG 3′; *adamts5*-qF: 5′ GTC ACA TGA ATG ATG CCC AC 3′*; adamts5*-qR: 5′ CAA ATG GCA GCA CCA ACA TA 3′; *adamts8*-qF: 5′ ATC ACC GTG AGG ATG TGG TT 3′,* adamts8*-qR: 5′ CAA GAG GTT TGT GTC CGA GG 3′; *adamts9*-qF: 5′ TGT GGT GTT GGA GTG ATG CAG AGA 3′,* adamts9*-qR: 5′ TCT GGC TTC AGA TCA GTG TGG CAT 3′; *adamts15*-qF: 5′ ACA CTG CCA TCC TCT TCA CC 3′, *adamts15*-qR: 5′ TCT TGG GGT CAC ACA TGG TA 3′. Quantitative PCR (qPCR) primers were designed over introns. iTaq Universal SYBR Green Supermix is from Bio-Rad. The following cycling conditions were used with 10 ng of RNA: 95℃ for 30 s, followed by 40 cycles of amplification (95℃ for 5 s, 60℃ for 30 s, 55℃ for 60 s, read plate), and a melting curve analysis. Relative quantities of RNA were determined using the ΔΔ-CT method. A minimum of *n* = 3 experiments (each in triplicate) was run for each gene. All data were normalized to *gapdh* levels.

### In Situ Hybridization

*In situ* hybridization (ISH) was performed on 16 µm coronal cryosectioned tissues as previously described. The antisense riboprobe generation of full length of *adamts4* and *syt1* were described previously (Su et al., 2010; Levy et al., 2015). Briefly, riboprobes were synthesized using digoxigenin (DIG)-labeled UTP (Roche, Mannheim, Germany) and the MAXIscript In Vitro Transcription Kit (Ambion, Austin, TX, USA). Probes were hydrolyzed to 500 nt. Sagittal brain sections were prepared and hybridized at 65℃ as previously described (Su et al., 2010), and bound riboprobes were detected by horseradish peroxidase (POD)-conjugated anti-DIG antibodies and fluorescent staining with Tyramide Signal Amplification (TSA) systems (PerkinElmer, Shelton, CT, USA). Images were obtained on a Zeiss Axio Imager A2 fluorescent microscope or a Zeiss Examiner Z1 LSM 700 confocal microscope. A minimum of three animals per genotype and age was compared in ISH experiments.

## Results

### Loss of WFA-Labeled PNNs in the Cerebral Cortex and Hippocampus of Collagen XIX-Deficient Mice

**Figure 1.**
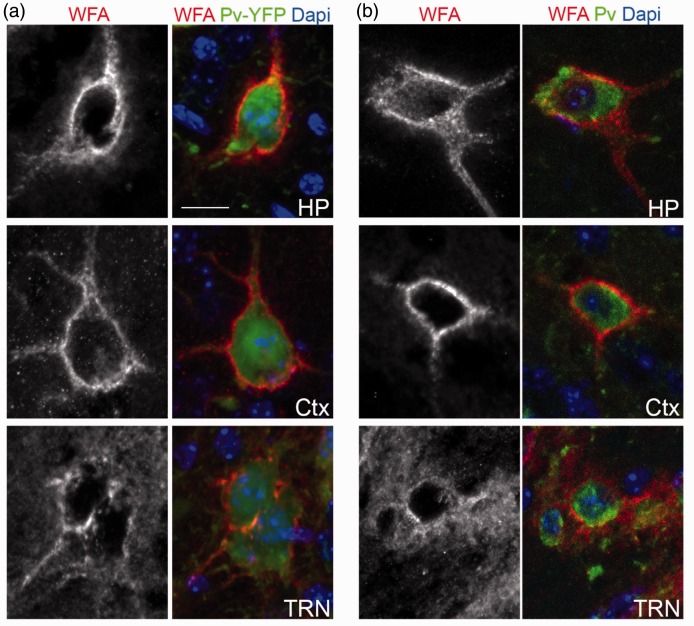
PNNs ensheath the cell bodies of Parv-expressing neurons in adult mouse brain. (a) WFA-labeled PNN were imaged in hippocampus (HP), cortex (Ctx), and the thalamic reticular nucleus (TRN) of P56 *parv-cre::thy1-stop-yfp15* transgenic reporter mice. Parv-expressing interneurons are genetically labeled with YFP in these mice. (b) WFA-labeled PNN were imaged in hippocampus, cortex, and the thalamic reticular nucleus (TRN) of P56 wild-type mice. Parv-expressing interneurons were labeled by IHC. Scale bar = 20 µm.

**Figure 2.**
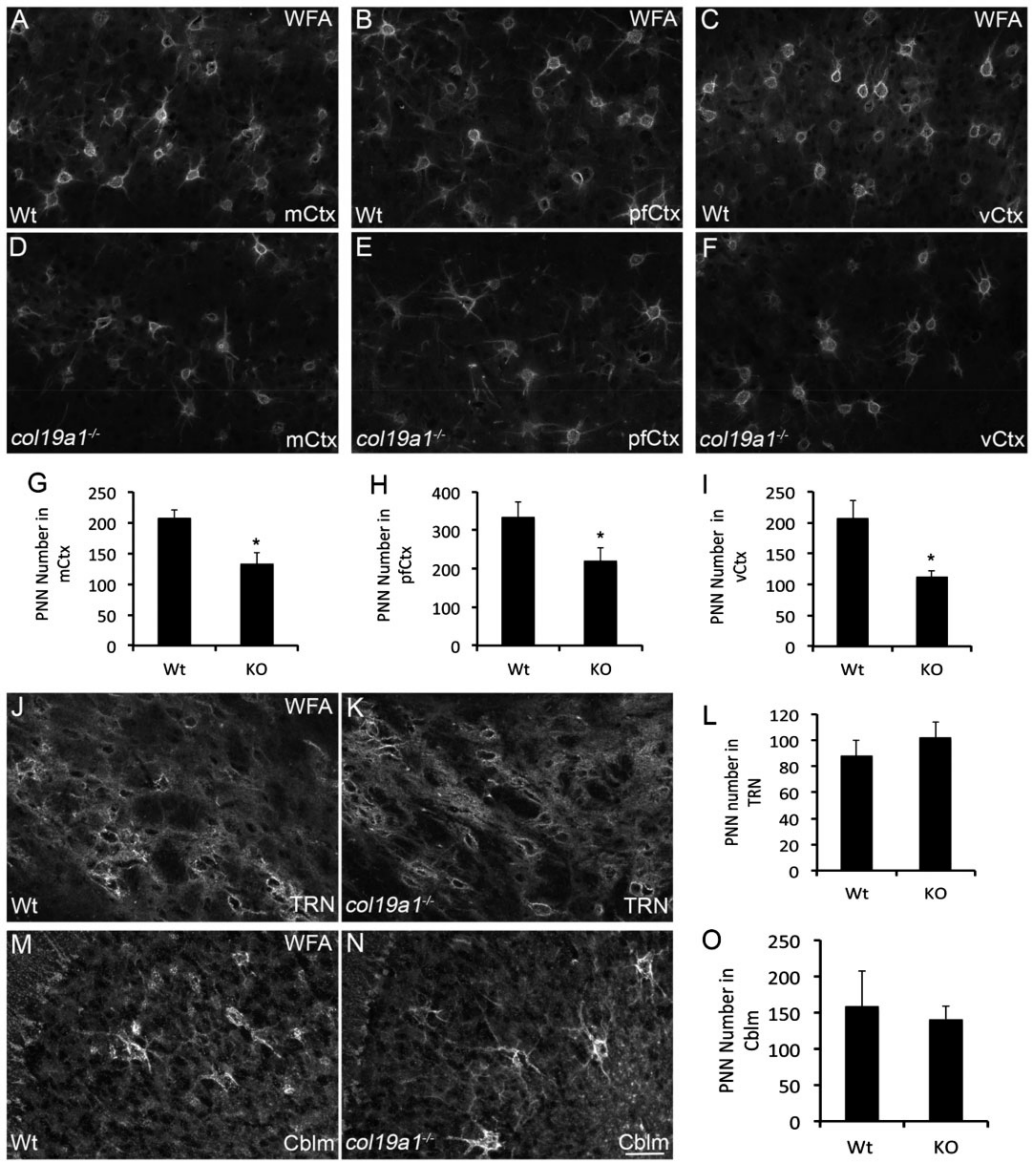
Loss of WFA-labeled PNNs in collagen XIX-deficient cerebral cortex. WFA-labeled PNN in P56 wild-type (WT; (a)–(c)) and *col19a1^−^^/^^−^* mutant (KO; (d)–(f))—motor cortex (mCtx; (a) and (d)), prefrontal cortex (pfCtx; (b) and (e)), and primary visual cortex (vCtx; (c) and (f)). Quantification of WFA-labeled PNN number in P56 WT and KO mCtx (g), pfCtx (h), and vCtx (i). Data represent means ± standard deviation (SD). * indicates differs from littermate WT by *p* < 0.05 by Student’s *t*-test. WFA-labeled PNN in the thalamic reticular nucleus of P56 WT (j) and *col19a1^−^^/^^−^* mutant (k). (l) Quantification of WFA-labeled PNN number in P56 WT and KO TRN. Data represent means ± SD. Data were not statistically significant. WFA-labeled PNN in the cerebellum (Cblm) of P56 WT (m) and *col19a1^−^^/^^−^* mutant (n). (o) Quantification of WFA-labeled PNN number in P56 WT and KO Cblm. Data represent means ± SD. Data were not statistically significant. Scale bar in *N* = 50 µm for (a)–(f), (j), (k), (m), and (n).

We previously reported that collagen XIX is generated by subsets of interneurons in the mammalian cerebral cortex (Su et al., 2016). To test whether collagen XIX is necessary for the formation or maintenance of PNNs, we assessed the presence of PNNs in several regions of the cerebral cortex of *col19a1^−^^/^^−^* mutant brains. PNNs were labeled with biotin-conjugated Wisteria floribunda agglutinin (WFA), a lectin that specifically binds N-acetylgalactosamines (Hartig et al., 1992; Figure 1). In adult wild-type mice, WFA labeled a large set of PNNs that surround the somas and proximal neurites of subsets of interneurons in all regions of cortex (Figure 2(a) to (c)). Similarly, in littermate *col19a1^−^^/^^−^* mutants WFA-labeling revealed PNNs in all regions of cortex and these specialized ECM assemblies appeared similar in morphology and size to those in controls (Figure 2(d) to (f) and data not shown), although the intensity of WFA-labeling appeared reduced compared to controls. However, we observed far fewer PNNs in the absence of collagen XIX (Figure 2(a) to (f)). We quantified PNN numbers in three regions of the cerebral cortex (pfCtx, mCtx, and vCtx) and discovered statistically significant reductions in WFA-labeled PNNs in all three regions of *col19a1^−^^/^^−^* brains (Figure 2(g) to (i)).

The distribution of WFA-labeled PNNs in mouse brains is far more extensive than the distribution of *col19a1*-expressing interneurons (Bruckner et al., 1994; Hartig et al., 1994; Bruckner et al., 2003; Su et al., 2010; Su et al., 2016). To test whether the loss of PNNs in *col19a1^−^^/^^−^* mutants was the result of a local role for collagen XIX or the result of general changes in mice that globally lack collagen XIX, we assessed PNNs in the thalamic reticular nucleus (TRN). TRN is a region of ventral thalamus containing PNN-ensheathed inhibitory projection neurons (Figure 2(j)), but that lacks collagen XIX expression (Su et al., 2016). The number and density of WFA-labeled PNNs in control and *col19a1^−^^/^^−^* mutant TRN were indistinguishable (Figure 2(j) to (l)). Likewise, the number of PNNs in cerebellum (Cblm), another region of mouse brain that lacks *col19a1*-expressing cells (Su et al., 2010), appeared similar control and *col19a1^−^^/^^−^* mutant mice (Figure 2(m) to (o)). Taken together, these results suggest a local role for collagen XIX in the assembly or maintenance of WFA-labeled PNNs.

**Figure 3.**
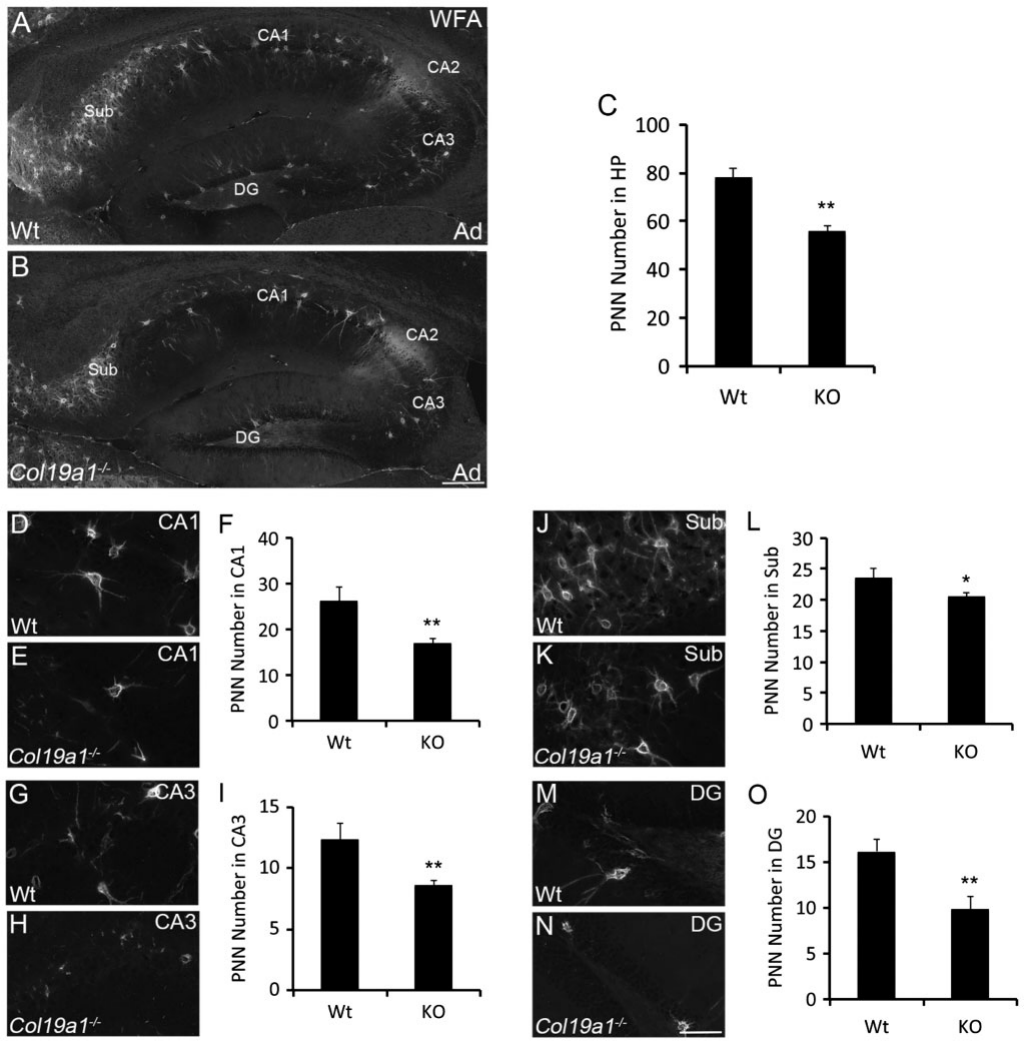
Loss of WFA-labeled PNNs in collagen XIX-deficient hippocampus. WFA-labeled PNN in hippocampus of P56 wild-type (WT; (a)) and *col19a1^−^^/^^−^* mutant (KO; (b)). (c) Quantification of WFA-labeled PNN number in P56 WT and KO hippocampus. Data represent means ± standard deviation (SD). ** indicates differs from littermate WT by *p* < 0.0001 by Student’s *t*-test. Distribution of WFA-labeled PNNs was assessed in subregions of P56 WT ((d), (g), (j), and (m)) and *col19a1^−^^/^^−^* mutant ((e), (h), (k), and (n)) hippocampi: Regions of analysis included conru ammonis area (CA) 1 ((d)–(f)), CA3 ((g)–(i)), subiculum (Sub; (j)–(l)), and dentate gyrus (DG; (m)–(o)). Quantification of WFA-labeled PNN number in these subregions are shown in (f), (i), (l), and (o). Data represent means ± SD. * indicates differs from littermate WT by *p* < 0.05 by Student’s *t*-test. ** indicates differs from littermate WT by *p* < 0.0001 by Student’s *t*-test. Scale bar in (b) = 300 µm for (a) and (b) and in (n) = 50 µm for (d), (e), (g), (h), (j), (k), (m), and (n).

To confirm this hypothesis, we addressed whether the number of WFA-labeled PNNs was altered in the hippocampus of *col19a1^−^^/^^−^* mutant mice. The hippocampus, a region within the telencephalon and a component of the limbic system, contains an abundance of PNNs (Figure 3(a); Bruckner et al., 2003) and interneurons that generate collagen XIX (Su et al., 2010). Indeed, there was a statistically significant reduction in PNN number in *col19a1^−^^/^^−^* hippocampi (Figure 3(b) and (c)). The hippocampus can be subdivided into at least five different areas—cornu ammonis (CA) areas 1–3, the dentate gyrus (DG), and the subiculum (Sub). In control mice, we observed prominent WFA-labeled PNNs in all five of these regions, with the highest density in Sub and CA2 (Figure 3(a)). We observed significant reductions in PNN numbers in four of these areas of collagen XIX-deficient hippocampi: CA1, CA3, DG, and Sub (Figure 3(d) to (o)). It is noteworthy that we failed to detect a significant reduction in WFA-labeling in CA2 in the absence of collagen XIX (Figure 3(a) and (b)). Sparing of PNNs in CA2 of collagen XIX-deficient hippocampi may reflect the distinct molecular architecture and ECM composition of this region (Bruckner et al., 2003; Carstens et al., 2016; Dudek et al., 2016) and the relative lack of collagen XIX expression by cells in this region (Su et al., 2010).

### Loss of Acan-Labeled PNNs in *col19a1^−^^/^^−^* Cerebral Cortex and Hippocampus

**Figure 4.**
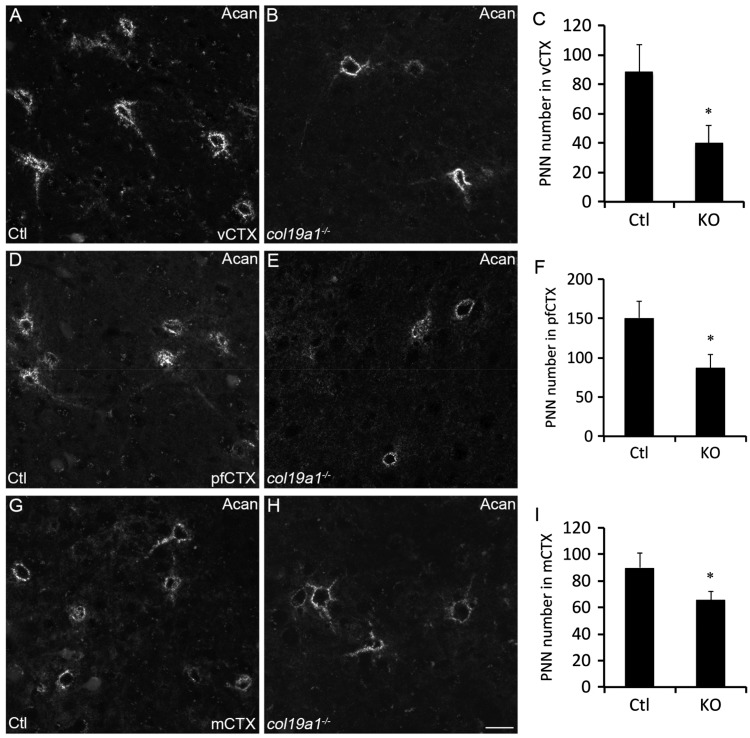
Loss of aggrecan-containing PNNs in collagen XIX-deficient cerebral cortex. Aggrecan (Acan)-immunolabeled PNNs in P56 wild-type (Ctl; (a), (d), and (g)) and *col19a1^−^^/^^−^* mutant (KO; (b), (e), and (h)). Images depict Acan-rich PNNs in primary visual cortex (vCtx; (a) and (b)), prefrontal cortex (pfCtx; (d) and (e)), and motor cortex (mCtx; (g) and (h)). Quantification of Acan-immunolabeled PNNs in P56 WT and KO vCtx (c), pfCtx (f), and mCtx (i). Data represent means ± standard deviation (SD). * indicates differs from littermate WT by *p* < 0.05 by Student’s *t*-test. Scale bar in (h) = 25 µm for (a), (b), (d), (e), (g), and (h).

**Figure 5.**
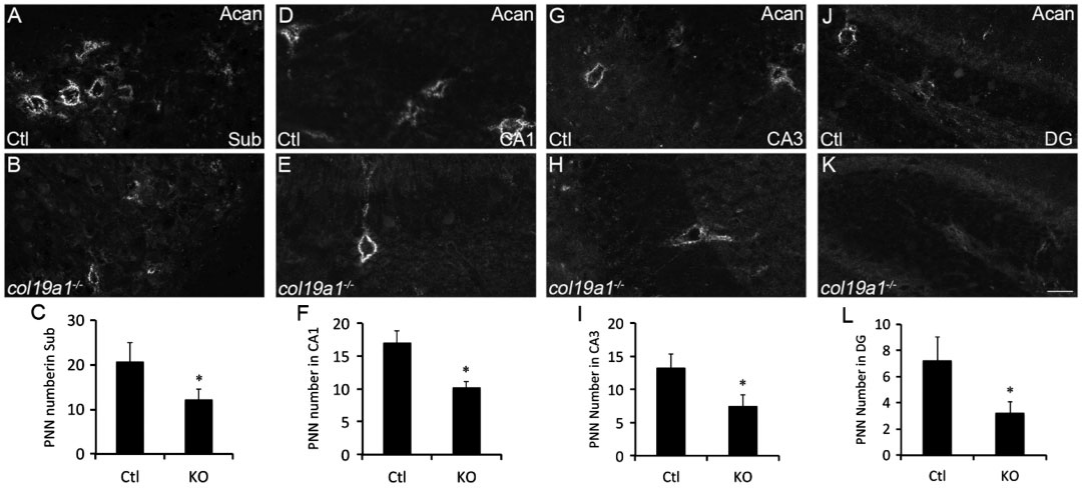
Loss of aggrecan-containing PNNs in collagen XIX-deficient hippocampus. Aggrecan (Acan)-immunolabeled PNNs in subregions of P56 wild-type (Ctl; (a), (d), (g), and (j)) and *col19a1^−^^/^^−^* mutant (KO; (b), (e), (h), and (k)) hippocampus. Images depict Acan-rich PNNs in Sub ((a)–(c)), CA1 ((d)–(f)), CA3 ((g)–(i)), and DG ((j)–(l)). Quantification of Acan-immunolabeled PNNs in P56 WT and KO in these subregions are shown in (c), (f), (i), and (l). Data represent means ± standard deviation (SD). * indicates differs from littermate WT by *p* < 0.05 by Student’s *t*-test. Scale bar in (k) = 25 µm for (a), (b), (d), (e), (g), (h), (i), and (k).

**Figure 6.**
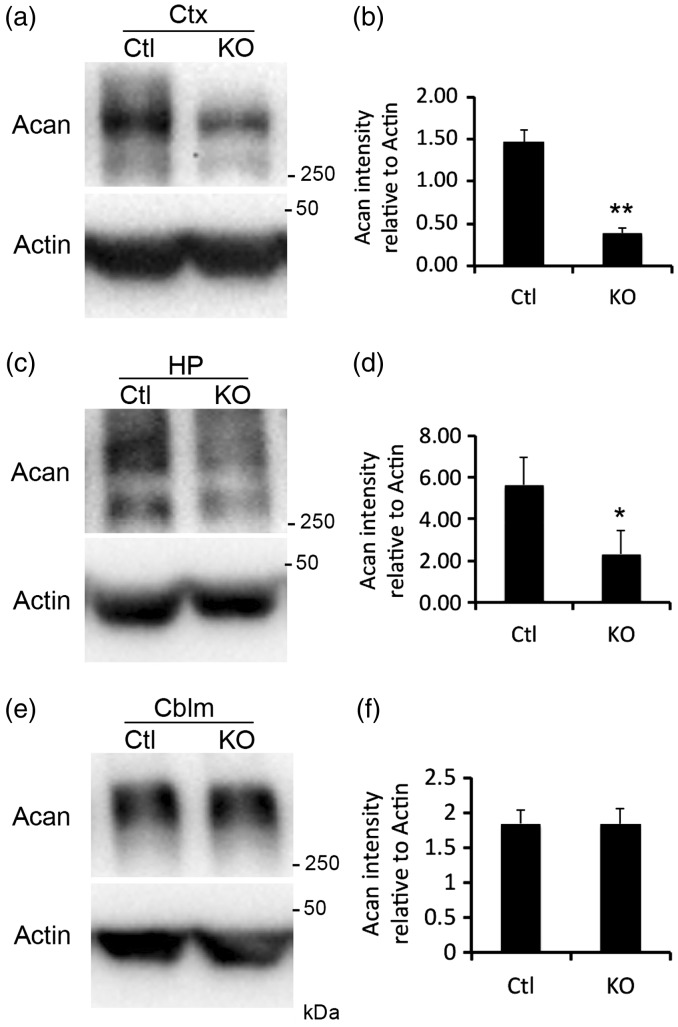
Reduced levels of aggrecan (Acan) in collagen XIX-deficient cerebral cortex and hippocampus. Western blots demonstrate reduced levels of Acan in protein extracts derived from P67 control (Ctl) or *col19a1^−^^/^^−^* mutant (KO) cortex (a), hippocampus (HP; (c)), and cerebellum (Cblm; (e)). Levels of Actin protein were used as loading controls. Quantification of levels of Acan detected by Western blot and normalized to Actin levels. Data shown are mean relative intensities normalized to Actin ± SD (*n* = 3). * indicates differs from littermate WT by *p* < 0.05 by Student’s *t*-test. ** indicates differs from littermate WT by *p* < 0.0001 by Student’s *t*-test.

To confirm the loss of PNNs in collagen XIX-deficient cerebral cortex and hippocampus (rather than the loss of WFA-binding sites with these PNNs), we took an alternative approach to label and quantify PNN number; we immunostained sections of control and *col19a1^−^^/^^−^* mutant brains with antibodies directed against Acan (Cat315; Matthews et al., 2002; Brooks et al., 2013), a lectican present in most PNNs (Zimmermann and Dours-Zimmermann, 2008). Acan-immunolabeled a similar, but not identical, population of PNNs as WFA in mouse cortex and hippocampus (Figures 4 and 5; Bruckner et al., 2003; Morawski et al., 2012). Similar to the results described above for WFA labeling, we detected a statistically significant reduction in the number of Acan-containing PNNs in pfCtx, mCtx, and vCTX (Figure 4) and in CA1, CA3, DG, and Sub (Figure 5) of *col19a1^−^^/^^−^* mutants. Based upon the reduced Acan-immunolabeling, we hypothesized that total telencephalic Acan levels were reduced in the absence of collagen XIX. To test this, protein extracts were isolated from the cerebral cortex or hippocampus of postnatal Day 67 (P67) control or *col19a1^−^^/^^−^* mutant mice. Western blots revealed a significant reduction in Acan levels in mutant cortex and hippocampus (Figure 6(a) to (d)). Reduced levels of Acan in collagen XIX-deficient mutants were highly specific to regions of mouse brain that normally generate collagen XIX, since levels appeared normal in cerebellum (Figure 6(e) and (f)). Taken together, these results demonstrate that the local loss of collagen XIX leads to significant reductions in both PNNs and PNN constituents in regions of brain that normally generated this unconventional collagen.

### Loss of Collagen XIX Leads to Altered Expression of Lectican-Degrading Proteases

**Figure 7.**
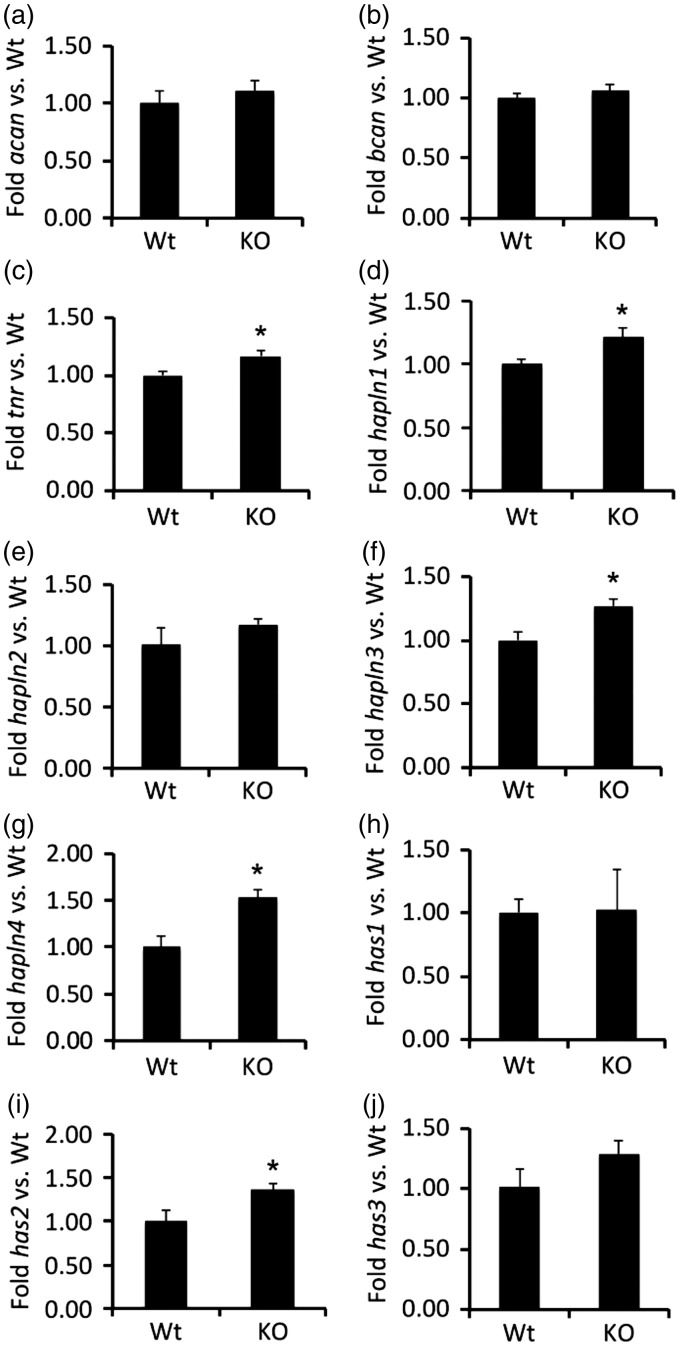
Alteration of PNN gene transcription in the absence of collagen XIX. Quantitative PCR (qPCR) analysis was performed on RNA extracted from P56 control (Ctl) or *col19a1^−^^/^^−^* mutant (KO) cortex. Levels of target gene expression were normalized to levels of *gapdh*. Data represent mean fold change (±SD) in mRNA expression in KO compared to levels in control samples. * indicates differs from littermate WT by *p* < 0.05 by Student’s *t*-test. Note that despite reduced levels of Acan protein levels in KO, qPCR revealed Acan transcription was unaltered in KO cortex. PNN genes analyzed: *acan* = aggrecan; *bcan* = brevican; *tnr* = tenascin R; *hapln1* = hyaluranonin and proteoglycan binding link protein 1; *hapln2* = hyaluranonin and proteoglycan binding link protein 2; *hapln3* = hyaluranonin and proteoglycan binding link protein 3; *hapln4* = hyaluranonin and proteoglycan binding link protein 4; *has1* = hyaluronan synthase 1; *has2* = hyaluronan synthase 2;* has3* = hyaluronan synthase 3.

The significant reduction in Acan protein led us to explore whether the loss of collagen XIX altered Acan transcription. To test this, RNA was isolated from the cerebral cortex of P56 control and *col19a1^−^^/^^−^* mutant mice and levels of *acan* mRNA were assessed by qPCR. We failed to detect significant alterations in *acan* mRNA levels in *col19a1^−^^/^^−^* mutant cortex (Figure 7(a)) suggesting the reduced Acan levels was not the result of altered transcription.

In fact, the same was true for other components of PNNs. We tested whether the mRNA expression of other integral components of PNNs, including other lecticans (like brevican), tenascin-R, HAPLN family members (HAPLN1-4), or Hyaluronan Synthases (Has1-3) were altered in *col19a1^−^^/^^−^* mutant cortex. Previous studies have revealed that partial or complete loss of any of these PNN constituents leads to reduced number or altered morphology of cortical PNNs (Weber et al., 1999; Bruckner et al., 2000; Carulli et al., 2010; Kwok et al., 2010). We found that the loss of Collagen XIX failed to reduce the transcription of mRNAs of any of these PNN constituents (Figure 7), and in several cases increased expression levels were detected (e.g., tenascin-R, HAPLN1, HAPLN3, HAPLN4, and Has2). This increased expression of PNN-associated genes may represent a homeostatic mechanism activated in collagen XIX-deficient mutants to counteract the loss of this collagen and PNNs.

**Figure 8.**
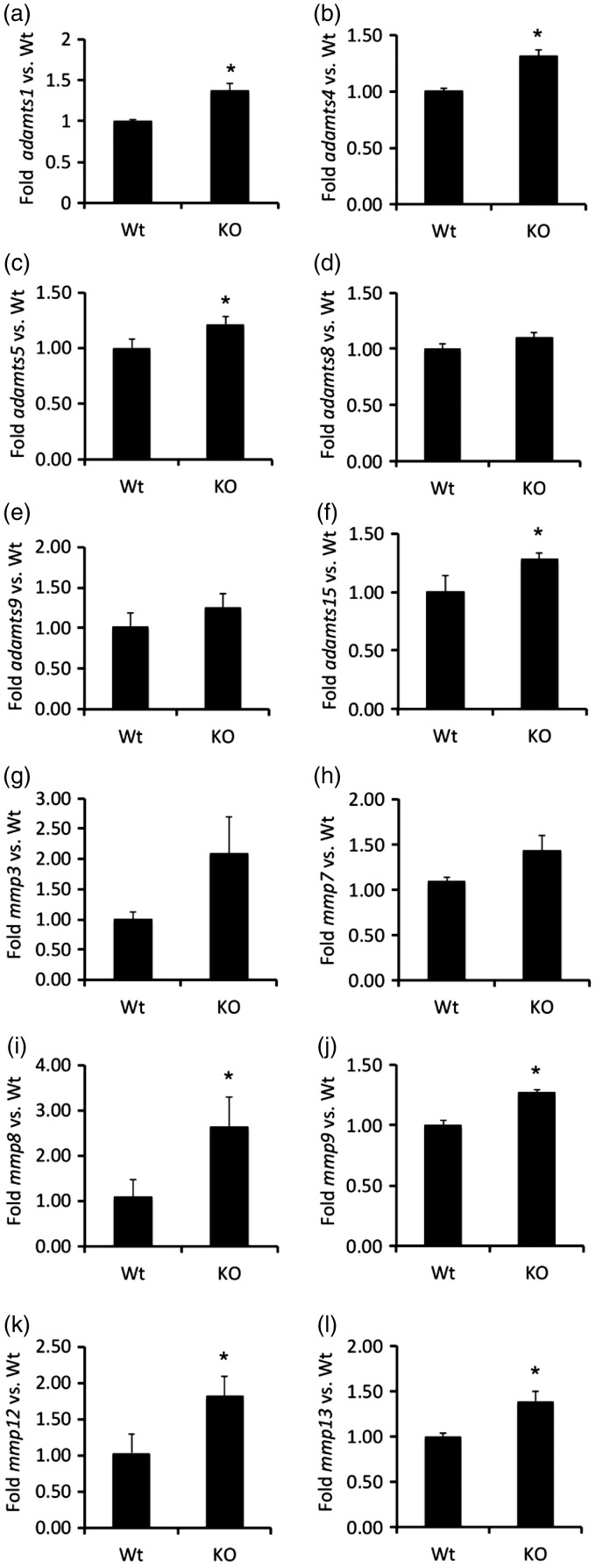
Alteration of protease gene transcription in the absence of collagen XIX. Quantitative PCR (qPCR) analysis was performed on RNA extracted from P56 control (Ctl) or *col19a1^−^^/^^−^* mutant (KO) cortex. Levels of protease gene expression were normalized to levels of *gapdh*. Data represent mean fold change (±SD) in mRNA expression in KO compared to levels in control samples. * indicates differs from littermate WT by *p* < 0.05 by Student’s *t*-test. Note that eight genes encoding PNN-degrading proteases from the ADAMTS and MMP families of proteases were upregulated in *col19a1^−^^/^^−^* mutant (KO) cortex.

The increase in expression of tenascin-R, HAPLN1, HAPLN3, HAPLN4, and Has2 in *col19a1^−^^/^^−^* mutant cortex failed to provide insight into the reduced levels of PNNs and Acan protein in these mutants. However, the increase production of these PNN constituents led us to speculate that collagen loss may also lead to an upregulation of extracellular proteases capable of degrading PNN components. PNN components are degraded by several families of metalloproteinases, including ADAMTSs and MMPs. Many ADAMTSs (including ADAMTS1, 4, 5, 8, 9, 11, and 15) are classified as lecticanases and not only have a high affinity and activity for cleaving Acan (Abbaszade et al., 1999; Tortorella et al., 1999; Tang, 2001; Porter et al., 2005), but are expressed in mouse cortex (Gottschall and Howell, 2015; Levy et al., 2015). Likewise, many MMPs are expressed in mouse brain and have been shown to cleave PNN constituents (Nagel et al., 2005; Rankin-Gee et al., 2015). We explored whether expression of 12 members of the ADAMTS and MMP families were altered in *col19a1^−^^/^^−^* mutant cortex. Eight of these proteases were significantly increased in collagen XIX-deficient cortex (Figure 8), including ADAMTS1, ADAMTS4, ADAMTS5, ADAMTS15, MMP8, MMP9, MMP12, and MMP13.

**Figure 9.**
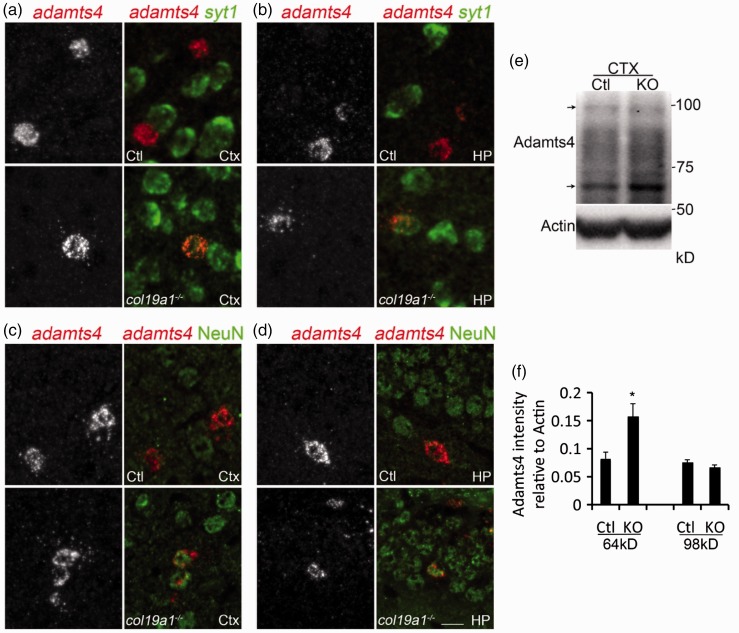
Ectopic neuronal expression of *adamts4* mRNA and increased active ADMATS4 peptides in the absence of collagen XIX. ((a) and (b)) *In situ* hybridization for *adamts4* and *syt1* mRNA in adult control or *col19a1^−^^/^^−^* mutant cortex (Ctx) and hippocampus (HP). Note the co-expression of *adamts4* and *syt1* mRNA in neurons in mutant Ctx and HP. ((c) and (d)) *In situ* hybridization for *adamts4* and IHC for NeuN in adult control or *col19a1^−^^/^^−^* mutant cortex (Ctx) and hippocampus (HP). Note the ectopic co-expression of *adamts4* and NeuN in neurons in mutant Ctx and HP. (e) Western blots of ADAMTS4 in protein extracts from control or *col19a1^−^^/^^−^* mutant Ctx. The pro- and active forms of ADAMTS4 are indicated with arrows (pro-form – 98 kDa; active form – 64 kDa). (f) Quantification of amounts of pro- and active forms of ADAMTS4 in control and *col19a1^−^^/^^−^* mutant Ctx. Data shown are mean relative intensities (normalized to actin) ± SD (*n* = 3). * indicates differ from controls by *p* < 0.05 by Student’s *t*-test. Scale bar = 20 µm.

One of the mRNAs increased in collagen XIX-deficient cortex encodes the most well-studied endogenous Acan-cleaving enzyme in the brain—ADAMTS4 (also called aggrecanase 1; Tang, 2001; Porter et al., 2005). We previously showed that *adamts4* mRNA expression was restricted to oligodendrocytes in the mouse cortex and hippocampus (Levy et al., 2015). Here, we discovered that *adamts4* mRNA becomes expressed in a subset of neurons in *col19a1^−^^/^^−^* mutant cortex and hippocampus (Figure 9(a) to (d)), suggesting that the significant increase in *adamts4* mRNA was the result of an upregulation of this gene in a cell-type that normally does not express it. Moreover, we assessed whether protein levels of ADAMTS4 were also altered in *col19a1^−^^/^^−^* mutant cortex. ADAMTS4 is produced as a pro-form (∼98 kDa) that undergoes cleavage to become a mature, active peptide (∼64 kDa)(Tortorella et al., 1999; Vistnes et al., 2014). In mutant and control cortex, we detected similar levels of the pro-form of ADAMTS4; however, increased levels of active ADAMTS4 were present in *col19a1^−^^/^^−^* mutant cortex (Figure 9(e)). Densitometry revealed that this increase in active ADAMTS4 was statistically significant (Figure 9(f)). Thus, these results reveal ectopic expression of *adamts4* mRNA in mutant brains and an increase in active ADAMTS4 peptides and therefore suggest that increased production and activity of Acan-degrading proteases contribute to the loss of PNNs in collagen XIX-deficient brains.

## Discussion

A recent study identified a novel micro-deletion at chromosome 6q12-13 in a cohort of patients with familial schizophrenia, a complex brain disorder characterized by alterations in cognitive function, acquisition of behaviors not observed in healthy individuals (such as hallucinations), and neglect and loss of apathy (Liao et al., 2012). This genomic region contains the coding sequence for collagen XIX. In support of the association of collagen XIX mutation or deletion with schizophrenia, we recently demonstrated that loss of collagen XIX leads to the acquisition of schizophrenia-related behaviors and seizures in mice (Su et al., 2016). These collagen-deficient mutant mice display a striking reduction in the formation of inhibitory axo-somatic synapses in the cerebral cortex and hippocampus (Su et al., 2010; Su et al., 2016), a class of synapse implicated in the mechanisms underlying schizophrenia, seizures, and other neurodevelopmental disorders (Gonzalez-Burgos et al., 2011; Lewis et al., 2011; Nakazawa et al., 2012; Wohr et al., 2015). Taken together, the loss of axo-somatic inhibitory synapses in *col19a1^−^^/^^−^* mutant mice and schizophrenic patients suggests a possible mechanism of how mutation, reduction or complete loss of collagen XIX may contribute to schizophrenia. However, there are other cellular and molecular alterations in the brains of patients with schizophrenia in addition to the loss of axo-somatic inhibitory synapses. One such alteration is the loss of interneurons (Zhang and Reynolds, 2002; Penschuck et al., 2006; Enwright et al., 2016). Previously we assessed interneuron number and distribution in cerebral cortex and hippocampus of collagen XIX-deficient brains and found no appreciable affect on interneuron number or distribution in the absence of this collagen in mice (Su et al., 2010; Su et al., 2016). Another alteration in patients with schizophrenia is PNN loss (Berretta et al., 2015; Bitanihirwe et al., 2016). Here, we set out to assess whether the loss of collagen XIX altered the number or distribution of PNNs. Our results demonstrate for the first time that the loss of a neuronally expressed collagen results in a significant reduction in the specialized lattice-like ECM that surround cortical and hippocampal interneurons. Therefore, these results identify a second mechanism by which the loss of collagen XIX may contribute to the development of neuropsychiatric disorders, such as schizophrenia.

### Does Collagen XIX Play a Direct role in PNN Assembly or Maintenance?

It is possible that interneuron-derived collagen XIX acts as a nucleating factor for PNNs. Outside of the nervous system, nonfibrillar collagens bind and anchor numerous ECM constituents into supramolecular assemblies (Ricard-Blum, 2011). Collagen XIX is a relatively recently identified, minor collagen (Myers et al., 1997; Sumiyoshi et al., 1997) and therefore the extent to which it binds other ECM components remains to be determined. In addition to its numerous collagenous domains, collagen XIX contains an N-terminal thrombospondin domain which may interact with other components of the brain ECM (Ricard-Blum, 2011). It also contains a C-terminal noncollagenous domain that interacts with a variety of RGD-dependent integrins (Oudart et al., 2015, 2016; Su et al., 2016) which are known to be expressed by interneurons in the cerebral cortex and hippocampus (Bi et al., 2001; Su et al., 2016). At present it remains unclear whether collagen XIX itself is present in PNNs. While collagen XIX is generated by subsets of cortical and hippocampal interneurons and PNNs preferentially ensheath interneurons in these brain regions, it is important to note that as many as 20 different classes of interneurons exist in these brain regions (Klausberger and Somogyi, 2008). Collagen XIX is generated by classes of interneurons that are defined neurochemically by their expression of calbindin, somatostatin, or neuropeptide Y and only a small fraction of these cells are ensheathed by PNNs (Su et al., 2010; Su et al., 2016). In contrast, PNNs preferentially ensheath classes of telencephalic interneurons that generate parvalbumin (Hartig et al., 1994) but only a small fraction of these cells appear to generate Collagen XIX (<10% of *col19a1*-expressing cells co-express Parv in cerebral cortex; Su et al., 2016). Therefore, while results presented here indicate a local role for collagen XIX in the assembly of PNNs, it is likely that many interneurons ensheathed by PNNs do not generate this collagen. An alternative possibility is that collagen XIX is secreted by one class of interneurons and aggregates around different classes of neighboring cells. This is the case for PNN constituents that are produced by glial cells (John et al., 2006; Giamanco and Matthews, 2012). And this type of paracrine mechanism appears to be how collagen XIX influences the development of inhibitory axo-somatic synapses in the cerebral cortex and hippocampus (Su et al., 2016). Of course, to test this hypothesis it will be essential to localize collagen XIX protein in PNNs *in vivo*, something that has not been possible with the current set of molecular tools to interrogate this collagen.

Although collagens may act as nucleating factors in basal laminas, it is entirely possible that the same is not true in PNNs and loss of PNNs may not be the *direct* result of the absence of this unconventional collagen. This possibility is supported by transcriptional changes of extracellular metalloproteinases known to degrade PNN constituents in *col19a1^−^^/^^−^* mutants. Many proteases upregulated in the cerebral cortex of *col19a1^−^^/^^−^* mutant mice are known to be generated by cortical interneurons that are coated by PNNs (Levy et al., 2015). So how might the loss of this collagen alter MMP or ADAMTS expression? A likely possibility is that this collagen plays a direct role in regulating transcription factors that act upstream of extracellular protease genes. Certainly the influence of ECM in regulating transcription in this fashion has been well documented outside of the brain (Bissell and Barcellos-Hoff, 1987; Spencer et al., 2007). Alternatively, the loss of collagen XIX is known to lead to molecular or cellular changes in the telencephalon (Su et al., 2010; Su et al., 2016) that may lead to altered protease production. While the number and distribution of PNN coated interneurons does not appear to change in *col19a1^−^^/^^−^* mutant brains, synapses onto these cells are altered in these mutants (Su et al., 2016). Perturbing connectivity in the brain is known to lead to alterations in protease production (Zhang et al., 1998; Jourquin et al., 2003), just as perturbing protease expression can alter connectivity (Huntley, 2012). In addition to structural changes in circuitry, the acquisition of behavior phenotypes and seizures in *col19a1^−^^/^^−^* mutant mice may lead to altered extracellular protease production. Prolonged seizure induction upregulates MMP and ADAMTS expression and the subsequent loss of PNNs in rodent models (Rankin-Gee et al., 2015). Likewise, an upregulation of ADAMTS15, which degrades Acan and other chondroitin sulfate proteoglycans and which we show is upregulated in cortex of *col19a1^−^^/^^−^* mutant mice, is upregulated in the hippocampi of patients suffering from medial lobe epilepsy (Venugopal et al., 2012). Thus, it is possible that altered connectivity and activity in *col19a1^−^^/^^−^* mutant mice leads to changes in protease production and a loss of PNNs.

### A Role for Collagen XIX in Closing the Critical Period of Neural Circuit Development

We now report two morphological phenotypes in the telencephalon of mice lacking collagen XIX—the loss of axo-somatic, inhibitory synapses onto Parvalbumin-expressing interneurons (Su et al., 2016) and the loss of PNNs from this same class of interneurons. Our data indicate that alterations in the formation of inhibitory axo-somatic synapses appear well before the first PNNs begin to assemble in the developing cerebral cortex or hippocampus (Su et al., 2010; Su et al., 2016). However, it is very likely that these phenotypes are interconnected, since they involve the same cell type and both morphological phenotypes are associated with a variety of complex brain disorders. Relatively late in development and after a period of activity-dependent refinement of neural circuits, PNNs emerge and are thought to form a physical barrier to prevent either the loss of established axo-somatic connections or the formation of new connections (Wang and Fawcett, 2012). The emergence of PNNs coincides with the closing of the “critical period” in circuit development, a period in which neural activity and experience are critical for shaping neural circuit refinement. To test the link between PNNs, axo-somatic synapse and the critical period of circuit development, previous laboratories have enzymatically degraded PNNs in vCTX by delivering chondroitinaseABC, an enzyme that degrades CSPGs and PNNs. Experimental degradation of PNNs in vCTX led to both the re-emergence of ocular dominance plasticity (Pizzorusso et al., 2002) and enhanced motility of dendritic spines and increased structural and functional neuroplasticity in visual cortical circuits (de Vivo et al., 2013). Taken together with results here, and the temporal expression of collagen XIX in the postnatal brain (Sumiyoshi et al., 1997; Su et al., 2010; Su et al., 2016), we posit that loss of collagen XIX may stall the brain in an immature, plastic state and that its expression may contribute to the closing of the critical period in the developing cortex. While total loss of collagen XIX is detrimental due to the emergence of schizophrenia-related behaviors and seizures, our results raise the intriguing possibility that local manipulation of this collagen may be a useful therapeutic approach to modulate critical periods of cortical plasticity.
